# Nasal Hemorrhage

**Published:** 1886-12

**Authors:** 


### NASAL HEMORRHAGE.

We extract the following practical suggestion from the *Edinburgh Medical Journal:* In persistent hemorrhage from the nasal cavity, plugging the posterior nares should not be done until an attempt has been made to check the hemorrhage by firmly grasping the nose with the finger and thumb, so as completely to prevent any air from passing through the cavity in the act of breathing. This simple means, if persistently tried, will, in many cases, arrest the bleeding. The hemorrhage persists because the clot which forms at the rupture in the blood-vessel, is displaced by the air being drawn forcibly through the cavity in the attempt of the patient to clear the nostrils. If this air is prevented from passing through the cavity, the clot consolidates in position and the hemorrhage is checked.

